# Diagnostic Value of Serum Pancreatic Stone Protein in Gestational Diabetes Mellitus: A Prospective Cohort Study

**DOI:** 10.3390/jcm15072702

**Published:** 2026-04-02

**Authors:** Murat Polat, Sait Erbey, Mehmet Alican Sapmaz, Ömer Osman Eroğlu, Rabia Şeker

**Affiliations:** 1 Department of Obstetrics and Gynecology, Ankara Etlik City Hospital, 06170 Ankara, Turkey; saiterbey@gmail.com (S.E.); dr.alicansapmaz@hotmail.com (M.A.S.); omerosmaneroglu@gmail.com (Ö.O.E.); 2 Department of Medical Biochemistry, Ankara Etlik City Hospital, 06170 Ankara, Turkey; rabiatekinseker@hotmail.com

**Keywords:** gestational diabetes mellitus, pancreatic stone protein, human regenerating protein, biomarker

## Abstract

**Objective:** Pancreatic stone protein (PSP) is a secretory protein associated with systemic inflammation and β-cell stress. Given its reported association with type 2 diabetes mellitus, this study aimed to evaluate the relationship between serum PSP levels and gestational diabetes mellitus (GDM) and to assess its diagnostic performance. **Methods:** This single-center, prospective cohort study was conducted between June 2024 and May 2025. Eighty-four pregnant women at 24–28 weeks’ gestation were enrolled: 42 diagnosed with GDM according to Carpenter–Coustan criteria and 42 healthy controls. Serum PSP levels were measured using ELISA at the time of GDM diagnosis, and prior to treatment initiation. Group comparisons were performed using appropriate parametric and non-parametric tests. Diagnostic performance was evaluated by receiver operating characteristic (ROC) curve analysis. **Results:** Maternal weight was significantly higher in the GDM group (78.3 ± 10.0 kg vs. 68.4 ± 7.6 kg, *p* < 0.001). Serum PSP levels were significantly elevated in women with GDM compared to controls (8.89 ± 0.81 ng/mL vs. 7.72 ± 0.64 ng/mL, *p* < 0.001). Neonatal birth weight was also higher in the GDM group (3606 ± 507 g vs. 3355 ± 308 g, *p* = 0.008), while Apgar scores did not differ significantly. ROC analysis demonstrated an AUC of 0.883 (95% CI: 0.805–0.946, *p* < 0.001). At a cut-off value of 8.38 ng/mL, sensitivity was 76.2% and specificity was 85.7%. **Conclusions:** Serum PSP levels are significantly elevated in GDM and demonstrate good diagnostic performance. PSP may serve as a supportive biomarker reflecting β-cell stress in GDM, warranting further multicenter validation studies.

## 1. Introduction

Pancreatic stone protein (PSP), also known as regenerating protein or lithostatin, is a 16 kDa secretory protein belonging to the C-type lectin family, produced primarily in the pancreas and intestines [1,2]. This protein, which exhibits pro-inflammatory activity physiologically and triggers polymorphonuclear cell activation, is considered an important biomarker of the body’s response to systemic stress [3,4]. PSP is particularly noteworthy in clinical practice for the management of infection and inflammation processes. It has been proven to demonstrate higher diagnostic performance than established markers such as procalcitonin and C-reactive protein in the diagnosis of sepsis, and to assist in predicting the severity of sepsis and mortality risk [5].

The American Diabetes Association (ADA) describes gestational diabetes mellitus (GDM) as a condition in which glucose intolerance is first identified during the second or third trimester of pregnancy in individuals who did not have evident diabetes prior to becoming pregnant [6]. The prevalence of GDM varies greatly depending on population characteristics and diagnostic criteria and has been reported to range from 4% to 12% in studies [7]. Risk factors for GDM include obesity, low socioeconomic status, immigration, advanced maternal age, family history of diabetes, and smoking [8,9,10]. GDM is associated with several complications, including preterm birth, fetal macrosomia, shoulder dystocia, increased rates of operative delivery and caesarean section, neonatal hypoglycaemia and neonatal hyperbilirubinaemia [10]. There are currently two accepted methods for diagnosing GDM. The first is a two-step approach, in which patients are first screened with a 50 g oral glucose challenge test (OGCT), and those with plasma glucose levels above 140 mg/dL are then administered a 100 g oral glucose tolerance test (OGTT) [6]. The second method is a single-step 75 g OGTT [6].

In the pathophysiology of diabetes, PSP is directly related to beta cell health and regeneration. Studies indicate that PSP is a potential biomarker for endoplasmic reticulum (ER) stress occurring in beta cells [11]. It has been demonstrated that when beta cells undergo apoptosis during the diabetic process, the released particles can trigger PSP expression in neighboring cells, thereby generating a response aimed at preserving beta cell mass [11,12,13]. PSP levels in patients with Type 2 Diabetes Mellitus (T2DM) increase in parallel with the duration and severity of the disease, reaching the highest levels particularly in individuals with microvascular complications such as diabetic nephropathy [14,15].

Pregnancy is characterized by progressive physiological immune modulation and low-grade systemic inflammation, driven by placental hormones such as human placental lactogen (hPL), progesterone, and estrogen, as well as pro-inflammatory cytokines including interleukin-6 (IL-6), tumor necrosis factor-alpha (TNF-α), and C-reactive protein [16]. In women who develop GDM, this inflammatory milieu is further amplified, exacerbating peripheral insulin resistance and impairing β-cell compensatory responses [17]. PSP, secreted under conditions of endoplasmic reticulum (ER) stress and systemic inflammation, may represent a sensitive indicator of this dysregulated metabolic–inflammatory interface. Unlike conventional inflammatory markers, PSP specifically reflects β-cell stress responses, offering a mechanistic link between placental-driven inflammation, insulin resistance, and islet cell dysfunction particular to the gestational context.

A recent study has shown that PSP is significantly increased in pregnant women diagnosed with preeclampsia and HELLP syndrome compared to healthy singleton pregnancies [18]. There is only one study investigating the relationship between GDM and PSP, and this study showed that there was no statistically significant difference between PSP values measured in women with GDM who received diet or insulin therapy and PSP values in healthy pregnancies [19]. Given that PSP shows strong potential as an indicator for the early detection and progression of T2DM, further research into PSP’s role in pregnancy-related systemic inflammation conditions and beta cell dysfunction, particularly in the context of GDM pathophysiology, is important. This study aims to evaluate the relationship between PSP and GDM and its role as a new diagnostic biomarker.

## 2. Materials and Methods

### 2.1. Study Design

This study, designed as a single-center, observational, prospective cohort study, was conducted at Etlik City Hospital, a tertiary care center, between June 2024 and May 2025. In our study, GDM diagnosis was made according to the Carpenter–Cousan criteria [6]. Pregnant women between 24 and 28 weeks of gestation who underwent GDM screening, were diagnosed with GDM, and were healthy were invited to participate in the study. Patients with T2DM and type 1 diabetes, hypertension, or renal and hepatic insufficiency were excluded from the study. The study was conducted with 42 healthy pregnant women and 42 pregnant women with GDM who agreed to participate. Those with OGL < 140 mg/dL were included in the control group. Those with OGL results ≥ 140 mg/dL and 100 g OGTT results meeting the Carpenter–Cousan criteria for GDM diagnosis were included in the patient group.

The following information was collected from the pregnant women included in the study: age, height, weight, gravida, parity, gestational age, and OGL and OGTT results. A peripheral blood sample was taken to measure serum PSP levels prior to initiation of any treatment (i.e., before diet or insulin therapy was commenced). After delivery, the following information was recorded from the medical records of the pregnant women included in the study: newborn birth weight, 1 min and 5 min APGAR scores, whether the newborn had complications and, if so, the type of complication, whether the pregnant women had complications during delivery and, if so, the type of complication.

### 2.2. Sample Collection and Processing

A 5–10 mL peripheral blood sample was taken from patients prior to initiation of any treatment to measure serum PSP levels. The sample was then centrifuged to separate the serum fraction and stored at −80 °C. PSP was measured by using the enzyme-linked immunosorbent assay (ELISA) kit as per the protocol of commercial kit manufacturer instructions. Blood samples taken from patients were kept at room temperature for 20 min to complete the clotting process. They were then centrifuged at 3000 rpm for 20 min, and the resulting serum samples were separated and stored at −80 °C until the day of the study. The PSP levels were calculated from the obtained standard curve and the levels were expressed as ng/mL. The calculated intra-assay CV was <8%. The functional sensitivity was 0.029 ng/mL, and the Standard Curve Range was 0.05–20 ng/mL.

### 2.3. Ethical Approval

Ethical approval for the study has been obtained from the Ethics Committee of Ankara Etlik City Hospital (approval code: AESH-BADEK-2024-519; approval date: 12 June 2024). The study was conducted in accordance with the Declaration of Helsinki. Written informed consent was obtained from all participants.

### 2.4. Sample Size and Power Analysis

Sample size was determined a priori by power analysis. Based on an independent *t*-test design with an effect size of 0.8 (large), a significance level of α = 0.05, and a statistical power of 95%, the minimum required total sample size was calculated as 84 participants (42 per group).

### 2.5. Statistical Analysis

The age, height, weight, gravida, parity, gestational age, OGL and OGTT results, serum PSP level, newborn birth weight, 1 and 5 min APGAR scores, whether the newborn had complications and, if so, the type of complication, and whether the participants had complications during delivery and, if so, the type of complication were recorded and then analyzed using SPSS 25.0.

The distribution characteristics of continuous variables were assessed using the Shapiro–Wilk test. Continuous variables showing a normal distribution were presented as mean ± standard deviation. Although non-parametric tests were used for comparisons of variables not showing a normal distribution, the data were reported as mean ± standard deviation in order to increase comparability in the study. In comparisons of two groups, normally distributed continuous variables were analyzed using the independent samples *t*-test, whereas the Mann–Whitney U test was preferred for continuous and ordinal variables that did not follow a normal distribution. Differences in categorical variables between groups were assessed using the chi-square test.

The ability of serum PSP levels to distinguish between patient and control groups was evaluated using Receiver Operating Characteristic (ROC) analysis. The area under the ROC curve (AUC) and 95% confidence interval were calculated. Sensitivity, specificity, positive and negative predictive values (PPV, NPV), and positive and negative likelihood ratios (LR^+^, LR^−^) were calculated for the determined cut-off value. All statistical tests were considered statistically significant at a two-tailed *p* < 0.05 value. Data were analyzed using IBM SPSS Statistics version 25.0 (IBM Corp., Armonk, NY, USA).

## 3. Results

A total of 84 cases were included in the study. The cases were divided into two groups: control and patient. Table 1 shows the values for age, height, weight, gravida, parity, and gestational week at blood sampling for both groups. No statistically significant differences were found between the control and patient groups in terms of age and height (*p* values 0.312 and 0.436, respectively). The mean body weight of the patient group (78.3 ± 10.0 kg) was significantly higher than that of the control group (68.4 ± 7.6 kg) (*p* < 0.001). No significant difference was observed between the groups in terms of gravida and parity (*p* values were 0.508 and 0.810, respectively). No significant difference was observed between the control and patient groups in terms of the gestational week at which blood samples were taken (*p* = 0.781) (Table 1).

Table 2 shows the OGCT, 100 g OGTT, serum PSP levels, and neonatal outcomes for the groups. The OGCT value was significantly higher in the patient group (169.3 ± 15.8 mg/dL) compared to the control group (116.1 ± 15.2 mg/dL) (*p* < 0.001). Since 100 g OGTT measurements were only available in the patient group, no statistical comparison could be made with the control group. The PSP level was 7.72 ± 0.64 ng/mL in the control group and 8.89 ± 0.81 ng/mL in the patient group, and there was a statistically significant difference between them (*p* < 0.001). The neonatal birth weight was 3606 ± 507 g on average in the patient group and 3355 ± 308 g in the control group, and the difference between them was statistically significant (*p* = 0.008). No significant difference was observed between the groups’ 1 min and 5 min APGAR scores (*p*-values were 0.085 and 0.363, respectively) (Table 2).

Table 3 shows the number of obstetric and neonatal complications observed in the groups. Vacuum-assisted delivery occurred in 1 person in the control group and in 2 persons in the patient group, while third-degree laceration was observed in only 1 person in the patient group (*p* = 0.501). Transient tachypnea of the newborn (TTN) was observed in 2 newborns in the control group and 6 newborns in the patient group, while clavicle fracture was observed in only 1 person in the patient group (*p* = 0.189). The participant with clavicle fracture was the same person who had vacuum-assisted delivery (Table 3).

**Figure 1:**
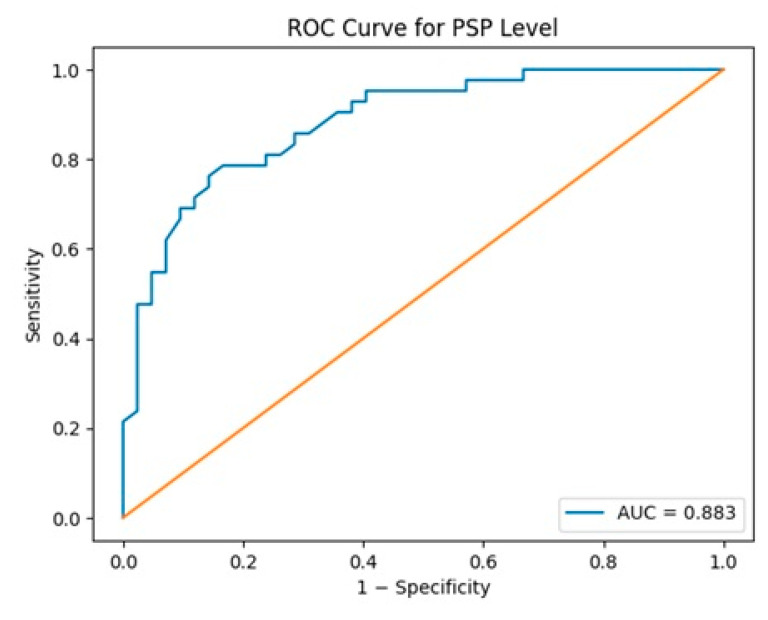
Receiver Operating Characteristic Curve for Serum PSP.

Table 4 presents the ROC curve results for PSP. The AUC calculated for PSP was 0.883 (95% CI: 0.805–0.946), sensitivity was 76.2%, specificity was 85.7%, PPV was 84.2%, NPV was 78.3%, and the best cutoff value was calculated as 8.38 ng/mL (*p* < 0.001). (Table 4) (Figure 1). The positive likelihood ratio was calculated as 5.33 and the negative likelihood ratio as 0.28 (Table 4).

## 4. Discussion

This study investigated the potential role of serum PSP levels measured between the 24th and 28th weeks of pregnancy in predicting GDM diagnosis and birth complications. The findings show that serum PSP values in pregnant women diagnosed with GDM are significantly higher than in healthy pregnant women.

Our study found a significant difference between the weight values of the groups. We explain the higher weight value in the patient group by the positive correlation between GDM risk and maternal weight [6,9,10].

Serum PSP levels were found to be significantly higher in the GDM group compared to the control group. This increase in serum PSP levels is consistent with the elevated serum PSP levels observed in T2DM patients compared to healthy controls [14,15]. In the study conducted by Vonzun et al., serum PSP levels in diabetic pregnant women did not show a significant difference compared to the control group. Vonzun et al. stated in their study that this was due to the strict control of GDM [19]. In our study, the PSP value was measured at the time of GDM diagnosis, before any treatment such as diet or insulin was initiated, and we believe this explains the difference from the results of Vonzun et al.

The pathophysiology of GDM involves a pregnancy-specific inflammatory milieu that is fundamentally distinct from that of T2DM. During normal gestation, placental hormones—particularly human placental lactogen (hPL) and progesterone—promote progressive maternal insulin resistance as a physiological adaptation to ensure adequate fetal nutrient supply. In women who develop GDM, this adaptation is exacerbated by elevated concentrations of pro-inflammatory cytokines including TNF-α, IL-6, and IL-1β, derived from both the placenta and hypertrophic adipose tissue [16,17]. These cytokines directly impair insulin receptor signaling and intensify the metabolic burden on β cells. PSP, which is upregulated under ER stress in β cells, may specifically reflect this pregnancy-amplified β-cell stress response. In contrast to T2DM—where chronic sustained hyperglycemia gradually exhausts β-cell capacity—GDM involves a more temporally compressed escalation of insulin resistance driven by rapidly rising placental hormones. This acute metabolic overload may sensitize β cells to ER stress in a pattern unique to pregnancy, leading to early PSP release. Future studies should simultaneously measure PSP alongside pregnancy-specific inflammatory mediators (IL-6, TNF-α, hPL) to delineate the mechanistic pathway driving PSP elevation in the gestational context and to clarify whether this pathway is distinct from that operative in T2DM.

Neonatal birth weight was found to be higher in the patient group compared to the control group. This finding is consistent with previous studies showing higher birth weight and fetal macrosomia in infants of mothers with GDM [9,10]. In our study, the absence of significant differences between groups in maternal and fetal complications contradicts the findings of studies in the literature. We believe that this situation may be due to the small number of patients in our study or the fact that patients maintained strict glycemic control from the diagnosis of GDM until delivery [9,10].

When we examine the ROC analysis results, we believe that the AUC value of 0.883 indicates that PSP has the potential to be a good biomarker for GDM screening. In particular, the sensitivity of 76.2% and specificity of 85.7% detected for the cutoff value of 8.38 ng/mL, along with LR^+^ 5.33 and LR^−^ 0.28 values, indicate that this protein has moderate-to-high diagnostic accuracy in clinical use. However, the literature generally refers to thresholds of LR^+^ ≥ 10 to strongly confirm the diagnosis and LR^−^ ≤ 0.1 to strongly rule out the disease [20]. In this context, it seems more appropriate to consider the PSP as an auxiliary biomarker in risk classification rather than a gold standard test on its own.

In current clinical practice, GDM screening relies on the OGTT, which, while widely validated, carries several practical limitations: it requires overnight fasting, a dedicated two-to-three-hour clinic visit with serial blood draws, and is poorly tolerated by a subset of pregnant women due to nausea or gastrointestinal discomfort. In this context, PSP—measurable from a single peripheral blood sample obtained at the time of routine GDM screening—may offer complementary clinical utility in specific scenarios. PSP could be particularly valuable in triaging high-risk patients who are unable or unwilling to complete a full OGTT. Furthermore, given that PSP specifically reflects β-cell endoplasmic reticulum stress and metabolic strain rather than glucose metabolism per se, it captures a distinct pathophysiological dimension of GDM that is not directly assessed by the OGTT. This biological complementarity suggests that PSP should not be viewed as a replacement for the OGTT, but rather as an adjunctive biomarker capable of enriching clinical risk stratification—particularly in cases where standard glucose testing is incomplete, inconclusive, or poorly tolerated.

The limitations of this study include its single-center design, the relatively modest sample size (n = 84), and the absence of a formal independent validation cohort—a recognized gold standard for biomarker research. The present findings should be considered hypothesis-generating; external replication in larger multicenter prospective studies is required before clinical implementation can be considered. Additionally, PSP measurement was limited to a single gestational window (24–28 weeks), precluding assessment of its dynamic course throughout pregnancy. The strengths of our study are its prospective cohort design and the fact that serum PSP values were measured at the time of diagnosis, before treatment had begun.

## 5. Conclusions

In conclusion, this study demonstrated that serum PSP levels are significantly elevated in pregnant women diagnosed with GDM and that this protein can be used in GDM diagnosis with moderate-to-high sensitivity and specificity. PSP’s role as an early marker reflecting impaired beta cell function may offer a new perspective in GDM management. Although OGTT remains the gold standard for GDM screening, we believe that early detection of GDM risk with a single blood measurement such as PSP could help patients be monitored more closely and be directed toward lifestyle changes at an early stage. We also believe that PSP could be helpful in analyzing GDM risk in patients who cannot tolerate OGTT. To translate these findings into clinical practice, larger, multicenter, prospective studies covering different stages of pregnancy are needed.

## Figures and Tables

**Table 1:** Demographic and Anthropometric Characteristics.

Variable	Control (Mean ± SD)	Patient (Mean ± SD)	Test	*p*
Age (years)	29.8 ± 4.6	30.4 ± 5.1	Independent *t*-test	0.312
Height (cm)	161.9 ± 5.7	162.9 ± 5.8	Independent *t*-test	0.436
Weight (kg)	68.4 ± 7.6	78.3 ± 10.0	Independent *t*-test	<0.001
Gravida	2.05 ± 0.88	1.90 ± 0.82	Mann–Whitney U	0.508
Parity	0.74 ± 0.73	0.71 ± 0.77	Mann–Whitney U	0.810
Gestational week at blood sampling	25.1 ± 1.1	25.1 ± 1.1	Mann–Whitney U	0.781

**Table 2:** OGTT Values and PSP Levels and Obstetric Findings.

Variable	Control (Mean ± SD)	Patient (Mean ± SD)	Test	*p*
OGCT	116.1 ± 15.2	169.3 ± 15.8	Mann–Whitney U	<0.001
100 gr OGTT–0 h	-	103.6 ± 15.8	-	-
100 gr OGTT–1 h	-	194.7 ± 23.5	-	-
100 gr OGTT–2 h	-	169.2 ± 20.8	-	-
100 gr OGTT–3 h	-	128.6 ± 31.0	-	-
PSP level (ng/mL)	7.72 ± 0.64	8.89 ± 0.81	Independent *t*-test	<0.001
Neonatal Birth weight (gr)	3355 ± 308	3606 ± 507	Independent *t*-test	0.008
APGAR score (1 min)	7.93 ± 0.68	8.19 ± 0.71	Mann–Whitney U	0.085
APGAR score (5 min)	9.19 ± 0.67	9.31 ± 0.75	Mann–Whitney U	0.363

**Table 3:** Obstetric and Neonatal Complications.

Complication Type	Control (n)	Patient (n)	Test	*p*-Value
None	41	39	Chi-square	0.501
Vacuum-assisted delivery	1	2		
3rd degree perineal laceration	0	1		
**Neonatal Complication type**	**Control (n)**	**Patient (n)**	**Test**	***p*** **-value**
None	40	35	Chi-square	0.189
Tachypnea	2	6		
Clavicle fracture	0	1		

**Table 4:** ROC Analysis of PSP.

Parameter	Value	95% Confidence Interval
Optimal Cut-off Value (ng/mL)	8.38	–
AUC	0.883	0.805–0.946
*p*-value	<0.001	–
Sensitivity (%)	76.2	–
Specificity (%)	85.7	–
Positive Predictive Value (%)	84.2	–
Negative Predictive Value (%)	78.3	–
Positive Likelihood Ratio (LR+)	5.33	–
Negative Likelihood Ratio (LR−)	0.28	–

## Data Availability

The data supporting the findings of this study are available from the authors upon reasonable request.

## References

[1] Watanabe T., Yonekura H., Terazono K., Yamamoto H., Okamoto H. (1990). Complete nucleotide sequence of human reg gene and its expression in normal and tumoral tissues. The reg protein, pancreatic stone protein, and pancreatic thread protein are one and the same product of the gene. J. Biol. Chem..

[2] Graf R., Schiesser M., Reding T., Appenzeller P., Sun L.K., Fortunato F., Perren A., Bimmler D. (2006). Exocrine Meets Endocrine: Pancreatic Stone Protein and Regenerating Protein—Two Sides of the Same Coin. J. Surg. Res..

[3] Widjanarko N.D., Soetedjo N.N.M., Iryaningrum M.R., Arifin E.S., Alvianto S., Lionardi S.K., Iskandar A.F., Chandra K.A. (2025). Pancreatic stone protein as a novel biomarker of microvascular complications in type II diabetes mellitus: A systematic review and meta-analysis. Tzu Chi Med. J..

[4] Pugin J., Daix T., Pagani J.-L., Morri D., Giacomucci A., Dequin P.-F., Guitton C., Que Y.A., Zani G., Brealey D. (2021). Serial measurement of pancreatic stone protein for the early detection of sepsis in intensive care unit patients: A prospective multicentric study. Crit. Care.

[5] Eggimann P., Que Y.-A., Rebeaud F. (2019). Measurement of Pancreatic Stone Protein in the Identification and Management of Sepsis. Biomark. Med..

[6] American Diabetes Association (2019). 2. Classification and Diagnosis of Diabetes: Standards of Medical Care in Diabetes—2019. Diabetes Care.

[7] Behboudi-Gandevani S., Amiri M., Bidhendi Yarandi R., Ramezani Tehrani F. (2019). The impact of diagnostic criteria for gestational diabetes on its prevalence: A systematic review and meta-analysis. Diabetol. Metab. Syndr..

[8] Mutsaerts M.A.Q., Groen H., Buiter-Van der Meer A., Sijtsma A., Sauer P.J.J., Land J.A., Mol B.W., Corpeleijn E., Hoek A. (2014). Effects of paternal and maternal lifestyle factors on pregnancy complications and perinatal outcome. A population-based birth-cohort study: The GECKO Drenthe cohort. Human. Reprod..

[9] Di Cianni G., Volpe L., Lencioni C., Miccoli R., Cuccuru I., Ghio A., Chatzianagnostou K., Bottone P., Teti G., Del Prato S. (2003). Prevalence and risk factors for gestational diabetes assessed by universal screening. Diabetes Res. Clin. Pract..

[10] Lowe L.P., Metzger B.E., Dyer A.R., Coustan D.R., Hadden D.R., Hod M., Oats J.J., Persson B., Trimble G.E.R. (2010). HAPO Study Cooperative Research Group. Hyperglycemia and Adverse Pregnancy Outcome (HAPO) Study: An Overview. Gestational Diabetes During and After Pregnancy.

[11] Stone S., Abreu D., Mahadevan J., Asada R., Kries K., Graf R., Marshall B.A., Hershey T., Urano F. (2019). Pancreatic stone protein/regenerating protein is a potential biomarker for endoplasmic reticulum stress in beta cells. Sci. Rep..

[12] Eizirik D.L., Cnop M. (2010). ER Stress in Pancreatic β Cells: The Thin Red Line Between Adaptation and Failure. Sci. Signal.

[13] Wilkinson B., Gilbert H.F. (2004). Protein disulfide isomerase. Biochim. Et Biophys. Acta (BBA)—Proteins Proteom..

[14] Yang J., Li L., Raptis D., Li X., Li F., Chen B., He J., Graf R., Sun Z. (2015). Pancreatic stone protein/regenerating protein (PSP/reg): A novel secreted protein up-regulated in type 2 diabetes mellitus. Endocrine.

[15] Albadr A., Haddad N.S. (2023). Pancreatic Stone Protein/regenerating Protein (PSP/reg) as a Biochemical Marker for prediction of Microvascular Complications of Type 2 Diabetes Mellitus. AL-Kindy Coll. Med. J..

[16] Pantham P., Aye I.L.M.H., Powell T.L. (2015). Inflammation in maternal obesity and gestational diabetes mellitus. Placenta.

[17] Lappas M., Hiden U., Desoye G., Froehlich J., Mouzon S.H., Jawerbaum A. (2011). The Role of Oxidative Stress in the Pathophysiology of Gestational Diabetes Mellitus. Antioxid. Redox Signal.

[18] Brun R., Vonzun L., Cliffe B., Gadient-Limani N., Schneider M.A., Reding T., Graf R., Limani P., Ochsenbein-Kölble N. (2023). The Role of Pancreatic Stone Protein (PSP) as a Biomarker of Pregnancy-Related Diseases. J. Clin. Med..

[19] Vonzun L., Brun R., Gadient-Limani N., Schneider M.A., Reding T., Graf R., Limani P., Ochsenbein-Kölble N. (2023). Serum Pancreatic Stone Protein Reference Values in Healthy Pregnant Women: A Prospective Cohort Study. J. Clin. Med..

[20] Jaeschke R. (1994). Users’ Guides to the Medical Literature. JAMA.

